# Cellular Genomic Sites of Hepatitis B Virus DNA Integration

**DOI:** 10.3390/genes9070365

**Published:** 2018-07-20

**Authors:** Magdalena A. Budzinska, Nicholas A. Shackel, Stephan Urban, Thomas Tu

**Affiliations:** 1 Centenary Institute, University of Sydney, Sydney NSW 2050, Australia; m.budzinska@centenary.org.au (M.A.B.); n.shackel@unsw.edu.au (N.A.S.); 2 South Western Sydney Clinical School, University of New South Wales, Liverpool NSW 2170, Australia; 3 Gastroenterology, Liverpool Hospital, Liverpool NSW 2170, Australia; 4 Department of Infectious Diseases, Molecular Virology, Heidelberg Hospital University, D-69120 Heidelberg, Germany; Stephan.Urban@med.uni-heidelberg.de; 5 German Center for Infection Research (DZIF), Partner Site Heidelberg, D-69120 Heidelberg, Germany

**Keywords:** Hepatitis B Virus, hepatocellular carcinoma (HCC), next generation sequencing, inverse nested PCR, chromosomal instability, insertional mutagenesis, non-homologous end joining, cancer evolution, clonal expansion, viral persistence

## Abstract

Infection with the Hepatitis B Virus (HBV) is one of the strongest risk-factors for liver cancer (hepatocellular carcinoma, HCC). One of the reported drivers of HCC is the integration of HBV DNA into the host cell genome, which may induce pro-carcinogenic pathways. These reported pathways include: induction of chromosomal instability; generation of insertional mutagenesis in key cancer-associated genes; transcription of downstream cancer-associated cellular genes; and/or formation of a persistent source of viral protein expression (particularly HBV surface and X proteins). The contribution of each of these specific mechanisms towards carcinogenesis is currently unclear. Here, we review the current knowledge of specific sites of HBV DNA integration into the host genome, which sheds light on these mechanisms. We give an overview of previously-used methods to detect HBV DNA integration and the enrichment of integration events in specific functional and structural cellular genomic sites. Finally, we posit a theoretical model of HBV DNA integration during disease progression and highlight open questions in the field.

## 1. Introduction

Chronic infection by Hepatitis B Virus (HBV) is one of the major causes of liver cirrhosis and hepatocellular carcinoma (HCC) worldwide. Although there is a prophylactic vaccine to prevent virus infection, HBV currently infects ~290 million people [1], for whom there is no cure. People with chronic HBV infections are five to 100 times more likely to develop HCC compared to the general uninfected population [2,3]. This amounts to ~337,000 annual deaths from liver cancer secondary to HBV [4]. Moreover, while current treatments for chronic HBV (generally in the form of nucleotide analogues) reduce the development of cancer [5], HCC risk remains above normal during the first five years of therapy [6,7], particularly in patients with advanced liver disease. Surprisingly, the risk of HBV-associated HCC (though highly associated with chronic antiviral inflammation [8,9]) can persist after functional clearance of the virus infection [10], suggesting that other factors can drive hepatocarcinogenesis. These include chromosomal instability from past genotoxic injury, residual expression of oncogenic viral proteins, or the presence of pre-neoplastic genetic changes, all of which may be driven by integrated HBV DNA.

## 2. Hepatitis B Virus DNA Integration

**Figure 1:**
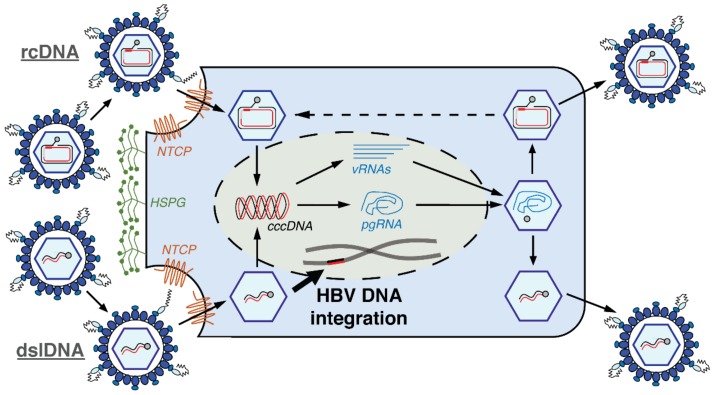
Replication cycle of Hepatitis B Virus (HBV) and its integration into the host genome. The nucleocapsid containing the relaxed circular DNA (rcDNA) (top half) or double-stranded linear DNA (dslDNA) (bottom half) HBV genome enters the cytoplasm via the sodium taurocholate co-transporting polypeptide (NTCP). In the nucleus, both forms can be converted into covalently closed circular DNA (cccDNA) which serves as the transcriptional template for all viral RNAs (vRNAs), including pre-genomic (pg)RNA. The pgRNA serves as the template for reverse transcription, which occurs within the nucleocapsid and results in rcDNA or dslDNA. The nucleocapsids can be then enveloped and secreted as virions. The intra-nuclear dslDNA HBV can integrate into the host cell genome at the site of double-stranded DNA breaks by non-homologous end joining (NHEJ). We have recently found that the reimport of dslDNA-containing nucleocapsids does not play a major role and that input HBV DNA is the main contributor of HBV DNA integration in in vitro models [16]. Figure adapted from [24].

Though sharing the same entry pathways as a productive HBV infection, the intracellular pathway that generates HBV DNA integration cannot produce new infectious virions (Figure 1). HBV virions exist as DNA-containing nucleocapsids enveloped by host-derived membranes that contain the HBV surface proteins. The intra-capsid double-stranded DNA genome can exist as one of two forms: one is a relaxed circular DNA (rcDNA) form; and the other (less frequent at 3–35% of virus particles [11]) is a double-stranded linear DNA (dslDNA) form. Both particles enter hepatocytes via attachment to heparan sulphate proteoglycans and subsequent interaction between the HBV large (L)-surface protein and the cellular sodium taurocholate co-transporting polypeptide (NTCP), a hepatocyte-specific bile acid transporter and cellular receptor for HBV [12,13]. Following membrane fusion (by an as-yet unknown mechanism), the nucleocapsid containing HBV DNA enters the cytoplasm and is transported to the nucleus [14].

Here the dsl- and the rcDNA forms can diverge. Both dslDNA and rcDNA can be converted by cellular enzymes into covalently closed circular DNA (cccDNA), which serves as the transcriptional template for all HBV messenger RNAs (mRNAs) and leads to virus amplification [15]. Sub-genomic viral mRNAs include those that give rise to the HBV X-protein (HBx) that acts as a transcriptional regulator and the HBV surface antigens (HBsAg) that can self-assemble into empty subviral particles. Pre-genomic RNA (pgRNA) is also transcribed from cccDNA and serves as the template for intra-capsid reverse transcription by the viral polymerase to result in rcDNA or dslDNA genomes. These nucleocapsids can be then enveloped and secreted as virions [14]. Alternatively, incoming intra-nuclear HBV dslDNA can integrate into the host cell genome [16] at the site of cellular double-stranded DNA breaks by non-homologous end joining (NHEJ) [17]. Next generation sequencing studies imply that HBV dslDNA represents the majority of the substrates for HBV DNA integration, though the junction sequence of a minority of integrations do not match up with the termini of the dslDNA form and suggest that other (unknown) HBV DNA forms may also be involved [18,19,20,21,22,23].

While the integrated form cannot generate new virions (as it cannot code for full-length pgRNA), the HBsAg open reading frame remains intact and can be actively expressed from the integrated form [25]. HBx with C-terminal truncations can also be theoretically produced from integrated HBV DNA, though the functionality of these proteins remains completely unknown.

## 3. Possible Functions of Hepatitis B Virus DNA Integration

Hepatitis B virus DNA integration is observed in all known hepadnaviruses, including the woodchuck and duck models of HBV infection [26,27,28], as well as chimpanzees [29,30] and humans [31,32,33] chronically infected with HBV. Despite this broad conservation, the functional consequences of integrated HBV DNA remain poorly understood.

Hepatitis B virus integration is associated with liver cancer development; 85–90% of HBV-related HCCs contain HBV DNA integrations [34], while they are less frequently detected in the non-tumour tissue (this is dependent on detection methods, as mentioned below). HBV integration has been reported (though not conclusively shown) to play an active role in hepatocarcinogenesis via multiple mechanisms, including: induction of chromosomal instability; disruption of cancer-associated genes by *cis*-mediated insertional mutagenesis; and formation of persistent templates for HBV gene expression (of either mutant or wild-type viral proteins). Hepatocellular carcinomas present with a high frequency of host genomic changes such as chromosomal rearrangements and copy number variations [18,21]. This higher genomic instability is reportedly induced by viral integration (e.g., by integrating into and disrupting the scaffold matrix associated regions used for cellular DNA-nuclear membrane interactions). Further, HBV integration adjacent to oncogenes or tumour suppressor genes (e.g., *hTERT*) has been observed, potentially altering gene function and their regulation by acting as a *cis* promoter/enhancer. Hepatitis B virus proteins (including PreS2 and HBx) can remain intact in the integrated form and can be expressed [35], and may act as transactivators in hepatocarcinogenesis. However, HBV DNA integration occurs early in the course of HBV infection, preceding the development of HCC by decades [36]. In vitro infection models have shown that HBV integration events can be found even <3 days post infection [16]. Thus, the true role of viral DNA integration in HBV-induced HCC remains unclear.

Another possible function of integrated HBV DNA is in inducing viral persistence by providing a stable reservoir for the transcription of the immunomodulatory HBsAg. A study in HBV chronically infected chimpanzees revealed that the majority of HBsAg transcripts are derived not from the cccDNA but from integrated HBV DNA [30]. Further, only a small reduction in HBsAg levels in HBeAg-negative animals was observed [30], consistent with human studies [37,38].

In conclusion, the functional impact of the HBV integration on the host genome is only partially understood and is a topic of growing importance. A major technical issue limiting the field is the detection of HBV DNA integrations, as an unbiased and highly-sensitive method for quantification of virus integration sites is still lacking.

## 4. Detection of Hepatitis B Virus Integration

Over the past decades, multiple methods have been used to detect virus integration into the host cell genome. Each of these methods has its distinct advantages and limitations (summarised in Table 1). While beginning with classical techniques such as Southern Blot hybridisation, recent development of high-throughput sequencing technologies (such as whole genome sequencing and RNA-Seq) has had a massive impact on the generation of large datasets, allowing for finer interrogation of the integration process and its implications.

Southern blot hybridisation using probes specific for HBV DNA was the initial method used to characterise integrated HBV DNA in HBV-related HCC and adjacent HBV-infected liver tissue [39,40,41,42,43]. Later, this analysis was extended to both HCC-derived cell lines [44] and liver tissue from HBV-positive cirrhotic patients without HCC [45]. However, the sensitivity of the method is very low, with a detection limit of 10^3^–10^5^ copies of the 3.2 kb HBV genome. Due to this, small clones (composed of <10^3^ cells) cannot be detected, causing a bias towards hepatocyte clones that have undergone extensive positive selection. Further, their copy number can only be quantified with poor precision through densitometry measurements.

The integrations of some highly-clonal samples were then painstakingly characterised by further cloning and direct sequencing. Targeting specific integration junctions in these samples (by cloning into plasmid vectors) allowed full analysis of the integrated HBV DNA fragment, in some cases showing complex rearrangements of the HBV genome and duplications in the cellular genome [45,47]. Though such approaches can provide detailed sequence information, they are not suitable for screening for a large range of unknown virus-cell junctions that exist in low copy numbers. More sensitive PCR-based methods have been developed to circumvent this limitation.

*Alu* PCR assays that use primers specific for HBV and *Alu* repetitive elements to amplify virus-cell DNA junctions have been used to detect HBV DNA integration in both HCC and non-tumour tissue of patients with HBV infection [48,49,50]. *Alu* sequences accounts for more than 10% of the human genome and are the most abundant repetitive elements [56]. Although the method is robust and requires small amounts of tissue, uneven distribution of *Alu* elements can prevent the amplification of the viral-human sequence as they might not be in the vicinity of *Alu*-repeats. Conversely, due to higher *Alu* density in some genomic regions, findings may be biased towards integrations located in specific functional regions (described below). In our experience [57], this technique has low specificity and sensitivity, with high amounts of *Alu-Alu* products being amplified in samples with low clonality (clones of <10^3^ cells). This technique also does not allow quantification of the integration junctions.

Inverse-nested PCR (invPCR) assays have been used to detect Duck Hepatitis B Virus (DHBV) DNA integrations from DHBV-infected duck liver [28], integrated Woodchuck Hepatitis B Virus (WHV) DNA in wild-infected woodchucks [26], HBV-infected chimpanzees [29], HBV integrations from human liver [31,32,33], and HBV-infected cell lines [16]. The major advantage of the method is high sensitivity, enabling detection of single copy virus-cell junctions [16,58]. Moreover, its selectivity and specificity allow identification of the exact virus-cell junction sequence and quantification of their absolute number. However, as this quantification occurs via end-point dilution, the assay is biased towards only detecting the virus-cell junctions in the largest cellular clones in the sample (though we recently showed that these do not appear to change during disease progression [59]). In addition, virus-cell DNA junctions are detected by use of restriction enzymes, which limits the detection of many (~90%) of integration junctions, though this can be accounted for using in silico analysis [16]. Finally, PCR amplification competition between products formed by HBV integration and products formed by defective HBV DNA may prevent reliable detection in some cell types (including primary human hepatocytes infected in vitro).

Recently, integration sites and the impact of HBV integration into the host cell genome have been explored using next-generation sequencing (NGS). The main advantages of NGS technology over other methods is the ability to sequence millions of reads in a single run and does not require prior information regarding the HBV or cellular sequences. Whole genome sequencing (WGS) gives the full coverage of the host genome and can be used to identify viral sequence aberrations such as mutations or structural rearrangements, which have been reported to accompany HBV DNA integration [60]. However, the cost of sequencing runs have necessitated limiting sequencing depths to at most 100×, which may result in a large number of HBV integration sites not detected, especially in normal tissue where there are smaller clones. While increased depth can be achieved with whole-exome sequencing (WES), this is accompanied by a corresponding loss of sequence coverage, as only coding regions of the human DNA genome and their flanking sequences are captured. With the cost of NGS continually falling, this is likely to change in the future, when the limiting factor will become the availability of computing power to analyse large datasets.

Whereas WES and WGS only give data on the DNA integration sites, RNA-seq can explore actively transcribed genes and allows for more sensitive prediction of sub-clonal HBV integration than WGS as it generates more reads covering the integration events. However, robust detection by RNA-seq is limited to HBV DNA integrations that have occurred in highly-expressed genes, are transcriptionally active themselves (driving high levels of transcription of HBx-cellular fusion genes from the HBx promoter), or have occurred in large cellular clones.

An additional weakness for NGS methods in general is using the appropriate computational tools, stringent controls, and ideal parameters and criteria to confidently identify an integration event. Due to the rarity of HBV DNA integrations (in terms of both number of copies per unique integration and the underlying integration rate), calling these events with certainty against the background of false positives (e.g., generated by PCR chimeras during library preparation) can be challenging. These potential artefacts should be ruled out with suitable experimental controls (e.g., spiking HBV DNA sequences into a non-infected control).

Finally, in the majority of all previous studies, the underlying biases of the detection method used (Table 1) are not taken into account during the interpretation of results. For example, if a tissue has more clonal heterogeneity (as is the case in non-tumour tissue when comparing to a HCC tumour [18,20]) fewer HBV integrations may be detected, simply due to the sensitivity of a particular technique for low copy number integration events. Moreover, if the method used has a bias (say, to detect integrations close to coding regions) a false enrichment for integrations in tumour tissues will be observed. Thus, it is important for researchers to be aware of or (more preferably) control for these detection biases in HBV DNA integration studies.

## 5. Site Specificity of Hepatitis B Virus Integrations

Using these molecular methods, HBV DNA integration has been observed throughout the genome. Recent studies have shown preferential integration in some genomic loci (outlined below). However, the mechanism underlying the selection of integration sites remains largely unknown. In general (Table 2), greater enrichment of integration into specific genes or features is seen in HCC tissue compared to non-tumour tissues (in which mostly random integration occurs).

### 5.1. Recurrent Genes

Hepatitis B virus DNA integrations in HBV-related HCC have been characterised for recurrent integration sites or preferential target genes. In general, more integration events have been observed in HBV-associated tumours compared to matched non-tumour tissue (86.4% vs. 30.7% [18] and 76.9% vs. 37.6% [20] in two separate studies), with only a small number of integrations shared by the tumour and non-tumour tissue in the same patient [18,20]. However, in a massive anchored parallel sequencing (MAPS) approach [61] significantly higher HBV insertional frequency was observed in adjacent non-tumour tissue compared to HCC tumour tissue (with 86% and 14% of the total integrations detected in non-tumour vs. tumour tissue, respectively), a difference which may be due to the different sensitivities of the corresponding methods. Additionally, more HBV integration sites in non-tumour liver tissues compared to tumour tissue were observed in RNA-seq studies [55].

Recurrent integration events have been frequently observed in the genes encoding human telomerase reverse transcriptase (hTERT), mixed-lineage leukaemia 4 (MLL4), and cyclin e1 (CCNE1) [18,20,22,23,62]. Interestingly, higher number of integrations in the *hTERT* gene is associated with a lower rate of *hTERT* promoter mutations, suggesting that they are mutually exclusive mutations [22,63].

Hepatitis B virus integration might have a direct effect on the expression of the host cell gene adjacent to the integrated HBV sequence and cause transcriptomic alterations such as HBV-human fusion transcripts. Although, the molecular mechanisms are unclear, they might be related to the effects on host cell promoters. Indeed, recurrent integrations are most frequently found in the promoter region of human *hTERT* gene [18,63,64,65], but have also been observed in *MLL4*, *CCNE1*, and *ALB* genes, in tumour samples but not in the matched non-tumour samples [18,66,67]. Many of these integrations have been shown to be associated with increased gene expression of the proximal cellular gene, presumably driven by viral elements.

Several HBV-human fusion transcripts generated as a consequence of HBV integration may result in chimeric protein expression with a potential oncogenic effect. Integration in the *SERCA1* gene in HCC tissue resulting in a HBx/SERCA1 fusion protein was reported to drive oncogenesis by inducing apoptosis [68]. Also, *HBx*/*MLL4* fusion transcripts have been observed in HCC patients and can be translated into short fusion proteins that suppress the expression of 11 cellular (potentially HCC-associated) genes in HepG2 cells [69]. Interestingly, HBV integration in the *FN1* gene seems to be specific to adjacent non-tumour liver tissue [18,55,61,70] and results in recurrent *HBV*-*FN1* fusion transcripts [70]. The purpose and effect of these fusion transcripts in non-tumour remains unknown.

The Sleeping Beauty (SB) transposon mutagenesis system has been used in mice for the discovery of key genes and pathways dysregulated in HCC by identifying common insertion sites. SB integrations are generally randomly distributed across the mouse genome in sense or antisense orientations [71]. Although the majority of SB insertions were unique, common insertion sites were present in multiple tumours from the same mouse (*n* = 14) [72]. Most of these genes have a predicted role as oncogenes or tumour suppressors and were significantly associated with Wnt/β-catenin and PKA/cAMP signalling pathways [72]. Moreover, high enrichment for genes that are implicated in metabolic processes were identified [73]. However, the SB transposon mutagenesis system generates many more integrations than HBV infection (~350 SB integrations compared to 1–10 HBV integrations per tumour) and the SB system requires additional stimuli for carcinogenesis (e.g., administration of di-ethyl nitrosamine, HBsAg overexpression), suggesting that these pathways may not reflect those driven by HBV DNA integration.

### 5.2. Recurrent Structures

#### 5.2.1. Telomeres

Telomeres, repetitive DNA regions (TTAGGG) at the ends of chromosomes, maintain genomic stability of the chromosomes and protect them from degradation and fusion events. Chronic liver injury, characterised by high liver cell turnover, accelerates telomere shortening, resulting in cell senescence or apoptosis, and chromosomal instability (CIS) that drives hepatocarcinogenesis [74,75]. Further, telomere length shows a gradual shortening during the multistep hepatocarcinogenesis in HBV-related disease [76]. HBV integration events have been reported to be significantly enriched in the proximity of telomeres in HCC DNA compared to paired non-tumour tissue [20]. Moreover, several novel HBV integration sites were identified in the DNA sequences of long noncoding RNAs related to telomere maintenance [61]. These all could potentially lead to telomere dysfunction, but the actual effect on hepatocarcinogenesis is not known.

#### 5.2.2. CpG Islands

Recent studies have shown significant enrichment of HBV integrations within CpG islands in tumours compared with non-tumour samples, with decreased frequency in genomic loci moving away from CpG islands [20]. However, the frequency of integration in these areas was not greater than expected by chance in RNA-seq datasets [55]. In our recent study, we found a slight (~2-fold) but significant enrichment within 10 kb of CpG islands in tissues from HBV-infected patients compared to a control in silico dataset [59]. However, we found no increased enrichment with liver disease progression, suggesting that HBV DNA integration near CpG islands may be an intrinsic feature of the integration process.

#### 5.2.3. Repetitive Regions (LINEs and SINEs)

Hepatitis B virus integration events have been observed within human repetitive regions, such as long interspersed nuclear elements (LINE), short interspersed nuclear elements (SINE) (including Alu repeats) and simple repeats (microsatellites) [61,66]. Furthermore, transcriptome sequencing of HBV-positive cell lines revealed HBV integration into LINE1 [54] and resultant expression of chimeric HBx-LINE1 RNA transcripts in ~23% of HBV-related HCCs, correlating with shorter survival time [54]. Further, HBx-LINE1 was shown to deplete cellular miR-122 and promote β-catenin signalling activation, E-cadherin reduction and cell migration [77]. However, the effect of HBx-LINE1 transcripts remains controversial after other authors failed to find this correlation in other cohorts [78].

#### 5.2.4. Transcriptionally Active Sites

Enrichment of HBV DNA integration into exons and promoters of coding genes has been observed in tumours compared with matched non-tumour tissue harbouring more integrations in introns [18,20,79]. However, there was no apparent prevalence of integrations in transcription factor binding sites or transcriptional start sites [20]. Our recent study showed no apparent prevalence in integration events into expressed genes, suggesting no preferential integration into transcriptionally active regions [20,59].

### 5.3. Recurrent Motifs

#### 5.3.1. Homology between Hepatitis B Virus and Cellular Sequences

Hepatitis B virus integrations occur at host DNA double-stranded breaks through both NHEJ and microhomology mediated end-joining (MMEJ; referred also as alternative NHEJ). Studies in the DHBV model [17] show that the majority of virus-cell junctions have little or no sequence homology shared with cellular DNA, therefore they probably follow the classical NHEJ pathway. Later, this was confirmed in both in vitro HBV infection models and ex vivo tissues from HBV-infected patients [16]. However, in about a third of integration junctions, short sequence homology (microhomology) between integrated HBV and cellular DNA at the site of the virus-cell junction has been observed [16,20,54,55,59], suggesting MMEJ as a potential repair mechanism in these cases.

#### 5.3.2. GC-Rich Regions

Hepatitis B virus DNA integrations have been found to integrated into the GC-rich regions of the host genome in PLC/PRF/5, a HBV-associated HCC-derived cell line [80]. It is important to note that SINE repeats (including *Alu*) accumulate over time in GC-reach genomic regions, and CpG islands have 60–70% GC content [81]. Further, two thirds of the protein-coding genes are concentrated in the GC-rich regions [82] and telomeric region of the chromosomes are GC-rich [83]. Thus, previously reported virus integration into repetitive regions, CpG islands, oncogenes, and telomeres suggests preferential integration proximal to cellular regions with high GC content. However, this hypothesis remains to be formally tested.

## 6. Current Model of Hepatitis B Virus DNA Integration during Disease Progression

**Figure 2:**
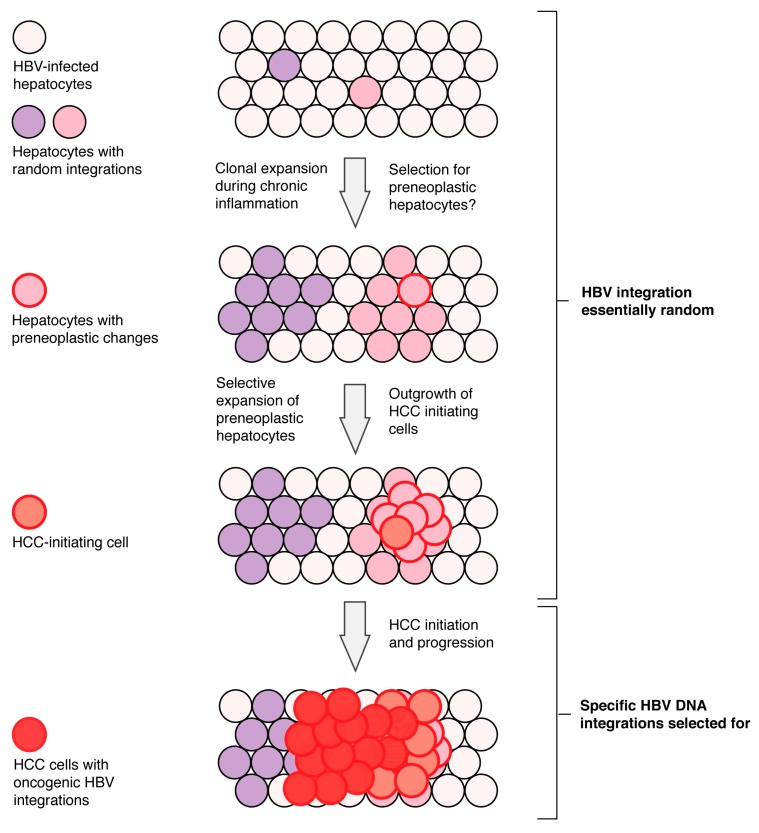
Model of HBV DNA integration during disease progression. Hepatitis B virus DNA integration into the host genome is random at the start of infection with tumour-associated specific enrichment of HBV integrations into regulatory regions (promoters) and HCC-associated genes (e.g., *hTERT*, *MLL4*) after the HCC initiation. Hepatocytes with random integrations clonally expand during the chronic antiviral inflammatory response. Over time, hepatocytes with some pre-neoplastic changes may be selected for and give a rise to HCC-initiating cells. Specific HBV DNA integrations are only selected for in late stages of HCC progression. Figure adapted from [9].

Together, the data suggests that HBV DNA integration into the host genome is random at the start of infection and in non-tumour tissue, but integration events into specific regulatory regions may be positively selected only during progression of HCC (Figure 2). In vitro [16] and animal models [28,84] show an integration rate of 1 in ~10^3^ cells within days of the initial HBV infection. During the disease progression of chronic HBV infection (particularly during HBeAg-seroconversion), hepatocytes with integrated HBV DNA undergo extensive selective clonal expansion (>10,000 cells). These selective advantages may play a role in the initiation of HCC [31,32,33] and could include DNA mutations or epigenetic changes that drive: hypersensitivity to growth factors; escape from immune recognition; or resistance to pro-apoptotic or -senescence signals. 

Hepatitis B virus DNA integration itself, however, is not a likely initiator of HCC. We and others have shown that the majority of HBV integration sites in non-tumour tissue are indistinguishable from random integration [18,20] and are not significantly enriched in any specific functional genomic regions during these clonal expansion phases [59]. This suggests that the majority of HBV integration events act as passenger mutations and do not play a role in HCC initiation (at least by *cis*-mediated mechanisms).

Instead, HBV DNA integration could play a role in HCC progression after initiation. Specific enrichment of HBV integrations into regulatory regions (promoters) and HCC-associated genes (e.g., *hTERT*, *MLL4*) has been observed in relatively small proportion of HBV-associated HCCs. In these, several of these reportedly pro-oncogenic integrations induce altered their gene expression in their downstream cellular genes, likely driven by the HBx promoter of the integrated HBV DNA. Moreover, HBV integration in tumour tissue tends to occur more frequently in regions with repetitive sequences, at chromosomal fragile sites, CpG islands, and transcriptional start sites. In summary, liver cancer development is a multistep process with accumulation of genetic alterations [85] and, given the data reviewed above, we believe that HBV integration likely plays *cis*-mediated oncogenic roles only late in tumour progression.

## 7. Open Questions Regarding Hepatitis B Virus Integration

### 7.1. What Proportion of Integrations Alter Cell Phenotype or Are Truly Pro-Carcinogenic? How and When during Disease Progression Do They Act?

In late stages of HCC, there is an enrichment of HBV DNA integrations in cancer-associated regions (e.g., *hTERT* promoters). However, our recent data shows that the cellular site profile of integrations is not altered during clonal expansion, suggesting that the majority of integrations do not contribute to cancer formation (at least by *cis*-mechanisms). This still leaves the possibility that a minority of integration events could be involved in hepatocarcinogenesis during early (pre-cancerous) stages. When detecting HBV DNA integrations in a patient tissue, an experimenter probably uses at most 100 mg of liver (one 10,000th of the whole liver) and so is unlikely to capture a rare event that might be the start of liver cancer. The lack of a good experimental system for HBV-associated HCC means that the field still remains unable to approach (in a physiologically-accurate manner) questions such as how HBV DNA integration can alter a hepatocyte to a pro-cancerous phenotype, when this change could occur, and how evolution towards HCC could be stopped. An immunocompetent small-animal model that supports the entire cycle of HBV replication and leads to human-like liver pathology (chronic inflammation, fibrosis, cirrhosis, and HCC) would be ideal for pursuing these questions.

### 7.2. What Controls the Frequency of Integration? Why Doesn’t Hepatitis B Virus Integration Occur More or Less Often?

Others groups and we have shown that integration events occur fairly consistently at a frequency of one per 10^4^ cells in both *in vitro* and *in vivo* models [16,28]. What exactly causes this consistent rate across the hepadnavirus family, why this integration rate seems to be conserved, and how it can be altered is unknown.

The levels of substrates of HBV DNA integration (cellular double-stranded breaks and HBV dslDNA in the inoculum) likely play a role in governing integration frequency, though this remains to be confirmed in human HBV infection models. In a DHBV in vitro infection model, it has been shown that increasing dslDNA levels in the inoculum increases integration rate. Moreover, in a HBV-expressing transgenic human cell line, increasing double-stranded breaks via peroxide-driven oxidative damage induced a 10-fold increase in detectable integrations. This has implications on the site of HBV integrations; it is possible that cellular regions with greater susceptibility to double-stranded breaks (e.g., CpG islands [20]) are also more susceptible to HBV integration. This interpretation stands in contrast to previously suggestions that HBV DNA integration causes genomic instability [61].

### 7.3. How Does Hepatitis B Virus Integration Occur Multiple Times in the Same Cellular Clone?

Related to the question above, several HCC tumours (and cell lines derived from them) show multiple integrations occurring within the same hepatocyte clone (e.g., four HBV copies in the PLC/PRF/5 cell line [86], at least three copies in the Huh-1 cell line [87]). Reconciling this observation with results seen in cell culture and infection models seems to set up a paradox.

First, we have found that integration rate does not appear to increase in productively infected cells (at least within a week after infection), but rather the majority of integration events occurs with the initial input infection [16]. If indeed integration occurs only during the initial infection event and if all cells are equally as likely to have an integration event, then with the relatively low integration rate of 10^−4^ integrations per cell (as described above), the probability of more than one integration event taking place in the same cell is highly improbable. Finally, super-infection exclusion (mediated by the virus L surface protein) prevents new NTCP-mediated infection of already-infected hepatocytes and has been observed in both DHBV [88] and HBV (Ni and Urban et al., manuscript in preparation) infection models. Together, these experimental observations suggest that each cellular clone should contain only a single HBV integration, despite clear evidence showing that multiple integrations occur.

There are multiple potential (non-exclusive) explanations of this apparent contradiction that remain to be experimentally tested. These include:

1. The lack of nuclear import of the de novo-generated mature nucleocapsids or superinfection exclusion is not absolute, but instead occurs at a slow rate in a chronically-infected hepatocyte. This allows multiple integrations to eventually accumulate within infected cells. This also predicts that the number of cccDNA per cell should also increase over long periods of time, which has not yet been observed. While only weeks-long in vitro models are available, no change in cccDNA levels has been observed after the initial formation [89]. Moreover, we have found that HBV mutants incapable of expressing HBcAg show no difference in cccDNA levels compared to wild-type after six weeks of infection cell [90], showing that nuclear import of nucleocapsids in infected cells is low in these models.
2. Not all cells are equally likely to contain integrations. In the liver cell population, there may be hepatocytes that have a susceptibility to HBV DNA integrations (e.g., cells with increased double-stranded breaks) in which multiple integrations could occur at the time of initial infection. Such an explanation could be explored using integration detection methods in single cells shortly after HBV infection.
3. Not all integrations express L-protein (e.g., due to epigenetic silencing, HBV truncations, mutations or lack of downstream poly-A in the integrated form), allowing re-infection of a cellular clone if cccDNA is cleared (e.g., through cell mitosis [91]. This mechanism would predict that only a single integration in a cellular clone (the most recent) would express the L-protein.

Addressing these potential explanations as testable hypotheses would not only help solve some inconsistencies observed between experimental infection systems and chronically-infected primary human tissues, but also expand our understanding of the viral-host dynamics of chronic HBV infections. This could open up new targetable variables to more efficiently disrupt viral persistence.

### 7.4. How Does the Structure of Integrated Hepatitis B Virus DNA Affect Viral Dynamics and Pathogenesis?

Only comparatively few integrated HBV genomes have been completely sequenced, and only from highly-clonal samples, such as HCC tissue or surrounding non-tumour tissue in late disease states. From these studies, the majority of fully-sequenced integrated HBV DNA have no viral genome rearrangements (including deletions, inversions, or duplications), despite common misconceptions to the contrary. Cloned HBV sequences from HCC [25,44,45] and surrounding non-tumour tissue, as well as WGS data [60], show that 80–90% of integrated dslDNA HBV genomes appear not to be highly rearranged (though contain extensive terminal truncations). The number of integrants with smaller alterations (e.g., small insertions/deletions) is more difficult to measure, due to the poor resolution of restriction fragment analysis (for Southern Blot and cloning analyses) and the excess amount of viral replicative intermediate DNA sequences leading to difficulties in mapping reads (for NGS-based analyses). The integrated genomes that do contain rearrangements have large deletions, insertions, duplications, inversions, and other complex structures. Interestingly, the loss of HBsAg PreS regions seems to be a common feature among these rearrangements [92]. While some rearrangements have been observed in the surrounding cellular DNA [92], these do not appear to be significantly different between tumour and non-tumour tissue [60].

Apart from the specific sequence of integrants, many other aspects regarding the structure of the integrated HBV DNA are still not known, including: (1) when these genomic rearrangements occur during infection (if the HBV DNA that integrates is already rearranged or if rearrangement takes place post-integration during clonal evolution); (2) if particular rearrangements are selected for during the process of clonal expansion or disease progression; (3) if specific cellular sites of HBV integration may be involved in controlling expression from the integrated form; and (4) if any clinical impact for the patient can be ascribed to the type of integration or its expression (e.g., involvement in HCC formation or HBV persistence).

Extending from the latter points, the clinical consequences of decoupling HBsAg and HBx expression from cccDNA persistence are of particular interest. HBsAg has known immunosuppressive functions [93,94,95] and its expression can be maintained in clonally expanded hepatocytes containing HBV DNA integrations in the face of antiviral responses targeting productively-infected cells. Thus, expression from integrated HBV DNA may play an important role in HBV persistence in the HBeAg-negative phase.

The consequences of persistent HBx expression are even more mysterious. While overexpression of HBx has been associated with multiple (possibly artefactual) pro-oncogenic pathways [96], its impact on HBV transcription and potential changes in viral dynamics with its decoupling with cccDNA has not been well-explored. It may be possible that transcriptionally-silent cccDNA (selected for by the antiviral immune response) could eventually be reinitiated by the HBx derived from integrated HBV DNA, thereby representing another persistence mechanism.

## 8. Summary

In conclusion, the unanswered questions about HBV-associated pathogenesis requires a better understanding of the virus and its host interactions. Both the understanding and recognition of HBV DNA integration in the pathogenic process are still poor, though increasing through the development of new detection techniques. These have revealed that HBV integration sites are mostly random with respect to the host genome from the beginning of infection until tumour initiation, but HCC progression is associated with enrichment in specific functional cellular genomic regions. The questions of if and how HBV DNA integration affects carcinogenesis (or indeed vice-versa) are still unanswered and require further research. Future studies in this field are therefore likely to lead to greater understanding and possibly to novel therapeutic targets for viral replication and persistence.

## Figures and Tables

**Table 1:** Summary of Hepatitis B Virus (HBV) DNA integration site detection methods.

Technique	Biases	Drawbacks	Advantages	Suitable Uses	Ref.
Southern blot	Dependent on restriction enzyme sites to resolve different integration events	Time consuming Technically demanding Low sensitivity (>10^3^ copies) No sequence information	Low cost Classical robust technique Absolute quantification possible (low precision)	Detecting presence of integrated HBV DNA in highly clonal samples	[39,40,42,43,44,45,46]
Direct cloning and Sanger sequencing	Dependent on restriction enzyme sites for cloning	Technically demanding Low-throughput	Complete integrated genome sequenced	Determining structure of integrated HBV genome in highly clonal samples	[45,47]
*Alu* PCR	Dependent on *Alu* sequences Biased towards larger clones	Multiple copies required for detection *Alu-Alu* products in low clonal samples No absolute quantification	Inexpensive Relatively simple	Detecting and sequencing integrated HBV DNA in clonal samples	[48,49,50]
invPCR	Dependent on restriction enzyme sites for detection Biased towards larger clones (as based on limiting dilution)	Time-consuming Technically demanding Only finds DNA sequence immediately adjacent to junctions	Absolute quantification High sensitivity (single copy) High specificity (detection of 1 in 10^6^ cells) Biases can be controlled for by in silico models	Detecting and quantifying rare HBV DNA integrations	[16,26,28,29,31,32,33]
WGS	Biased away from poorly mappable (e.g., transposon sequences) regions	Low-depth Cost No absolute quantification	Full genome coverage	Integration site detection in highly clonal samples	[18,19,20,21,22,23]
WES	Dependent on being in (or close to) coding regions	Coverage only of coding regions No absolute quantification	Greater depth than WGS	Integration site detection in coding regions	[51,52]
RNA-Seq	Biased towards more highly expressed genes	Coverage of expressed coding regions only No absolute quantification	Greater depth than WGS Data on transcriptional activity	Virus-fusion transcripts	[19,22,53,54,55]

invPCR, inverse-nested PCR; WGS, Whole Genome Sequencing; WES, Whole Exome Sequencing; RNA-Seq, RNA Sequencing.

**Table 2:** Enrichment of HBV DNA integrations into specific cellular regions and features.

Feature in Which Integration Occurs	Enrichment in HCC	Enrichment in Non-Tumour Tissue
Specific HCC driver genes	Yes, but minority of HCCs (TERT, MLL4)	FN1
Telomeres	Yes	No
CpG islands	Yes	Slight (~2-fold greater than expected)
Repetitive regions (e.g., LINEs and SINEs)	No, except one report [54]	No
Transcriptionally-active sites	Yes	No
Exons and Introns	Yes	Slight
Fragile sites	Yes	No
Promoter regions	Yes	Slight

HCC, Hepatocellular Carcinoma; LINEs, Long Interspersed Nuclear Elements; SINEs, Short Interspersed Nuclear Elements.
